# The Role of Autophagy and Apoptosis in Affected Skin and Lungs in Patients with Systemic Sclerosis

**DOI:** 10.3390/ijms241311212

**Published:** 2023-07-07

**Authors:** Vesna Spasovski, Marina Andjelkovic, Marina Parezanovic, Jovana Komazec, Milena Ugrin, Kristel Klaassen, Maja Stojiljkovic

**Affiliations:** Institute of Molecular Genetics and Genetic Engineering, University of Belgrade, Vojvode Stepe 444a, 11042 Belgrade, Serbia; marina.andjelkovic@imgge.bg.ac.rs (M.A.); marina.parezanovic@imgge.bg.ac.rs (M.P.); jovana.komazec@imgge.bg.ac.rs (J.K.); milena.ugrin@imgge.bg.ac.rs (M.U.); kristel.klaassen@imgge.bg.ac.rs (K.K.); maja.stojiljkovic@imgge.bg.ac.rs (M.S.)

**Keywords:** SSc, skin fibrosis, SSc-ILD, autophagy, apoptosis

## Abstract

Systemic sclerosis (SSc) is a complex autoimmune inflammatory disorder with multiple organ involvement. Skin changes present the hallmark of SSc and coincide with poor prognosis. Interstitial lung diseases (ILD) are the most widely reported complications in SSc patients and the primary cause of death. It has been proposed that the processes of autophagy and apoptosis could play a significant role in the pathogenesis and clinical course of different autoimmune diseases, and accordingly in SSc. In this manuscript, we review the current knowledge of autophagy and apoptosis processes in the skin and lungs of patients with SSc. Profiling of markers involved in these processes in skin cells can be useful to recognize the stage of fibrosis and can be used in the clinical stratification of patients. Furthermore, the knowledge of the molecular mechanisms underlying these processes enables the repurposing of already known drugs and the development of new biological therapeutics that aim to reverse fibrosis by promoting apoptosis and regulate autophagy in personalized treatment approach. In SSc-ILD patients, the molecular signature of the lung tissues of each patient could be a distinctive criterion in order to establish the correct lung pattern, which directly impacts the course and prognosis of the disease. In this case, resolving the role of tissue-specific markers, which could be detected in the circulation using sensitive molecular methods, would be an important step toward development of non-invasive diagnostic procedures that enable early and precise diagnosis and preventing the high mortality of this rare disease.

## 1. Introduction

Progressive systemic sclerosis (SSc), or scleroderma, is a rare systemic inflammatory disorder characterized by cellular inflammation, vasculopathy, skin fibrosis, and multiple internal organ fibrosis, including the gastrointestinal system, kidneys, and lungs [1]. SSc shares several common features with other rheumatological diseases, such as higher prevalence in women, a complex interaction of genetic and epigenetic factors as the mechanism of pathogenesis, and manifestations on several organs and tissues [2,3,4].

Skin changes present the hallmark of scleroderma and usually appear first. They are the indicators of clinical appearance, markers of internal organ involvement, and predictors of the disease outcome. Namely, more extensive skin involvement in SSc patients coincides with more severe internal organ manifestations and usually poor prognosis [5,6]. According to the classification criteria for SSc given by the American College of Rheumatology (ACR)/European League Against Rheumatism Collaborative Initiative (EULAR) from 2013, skin thickening is the first diagnostic criterion for SSc [7]. The modified Rodnan skin score (mRSS) is a ‘gold standard’ for diagnostics of skin changes. Its assessment is one of the first and most valuable clinical criteria for classifying SSc patients [7,8].

Interstitial lung diseases (ILD) are the most widely reported respiratory complications in SSc patients [9], and they are the reason that SSc has the highest disease-related mortality and substantial non-lethal complications among autoimmune diseases [10,11]. It has been reported that moderate-to-severe pulmonary fibrosis occurs in around 75% of patients with SSc [12]. Patients suspected or confirmed to have SSc should undergo a comprehensive clinical evaluation, which includes an assessment of respiratory symptoms and a high-resolution computed tomography scan (HRCT) of the chest [13]. ILD is also included in the ACR/EULAR joint classification criteria to identify SSc in individuals who do not have skin and joint manifestations of the disease [7].

Much effort has been made in order to elucidate the mechanisms relevant to the pathogenesis of SSc. The role of both genetic and environmental factors (toxic chemicals, infections or other physical traumas, intestinal dysbiosis, or dietary factors) have been investigated, providing the evidence that both have the influence on development and course of the disease [14,15,16]. Recent studies showed that the processes of autophagy and apoptosis could play a significant role in the pathogenesis and clinical course of autoimmune diseases [17,18]. Autophagy and apoptosis are associated in many aspects, including common triggering signals and executive pathways. Since autophagy can block apoptosis, and since both autophagy and apoptosis can lead to cell death, their regulation is highly orchestrated in homeostasis, and their role and effects in pathogenic conditions are the subjects of intensive research [19,20,21,22,23]. The results of GWAS studies, as well as custom SNP genotyping array targeting autoimmune disease loci, have shown enrolment of apoptotic genes, for instance, the *DNASE1L3* gene [24] and genes related to autophagy, such as autophagy-related 5 gene (*ATG5*) in SSc [25].

In this review, we aimed to summarize the current knowledge on the role of these two fundamental processes for cell survival in two organs that are mostly affected in systemic sclerosis—skin and lungs. Better understanding of molecular cascades enrolled in autophagy and apoptosis in SSc could be beneficial for the development of non-invasive and reliable diagnostic procedures, better and more accurate stratification of the patients, and development of therapeutics based on biomarkers for targeted therapy of affected organs.

## 2. Materials and Methods

Literature Search Strategy and Selection Criteria

This narrative review aimed to present the current knowledge of the processes of autophagy and apoptosis in the skin and lungs of patients with SSc. For that purpose, specific inclusion and exclusion criteria were used. 

Inclusion criteria were full-text research articles, narrative reviews, systematic reviews, and meta-analyses written in the English language. Only studies published in peer-- reviewed journals were analyzed. Publication date was not specified in the search, but the most recent literature was included and cited.

Exclusion criteria included non-English written articles, conference presentations, and expert opinions. Articles without an accessible abstract were also excluded.

The online databases PubMed and Science Direct were searched by two independent authors using the keywords “Systemic sclerosis” or “Scleroderma”, “skin involvement”, “skin fibrosis”, ”lung involvement”, “interstitial lung disease”, “nonspecific interstitial pneumonia“, and “idiopathic pulmonary fibrosis” combined with terms “autophagy”, “apoptosis”, “oxidative stress” in order to gain the most specific results. All incongruities were discussed between co-authors, until consensus.

## 3. Fibroblasts—Function and Transformation into Myofibroblasts in SSc

It is now accepted that endothelial and vascular damage is pivotal in the pathogenesis of SSc, and involves the activation of fibroblasts, which then overproduce collagen and other extracellular matrix (ECM) proteins [26,27]. Under normal physiological conditions, fibroblasts play a fundamental role in maintaining tissue homeostasis and wound healing by producing ECM components in a strictly controlled manner, which is silenced after the successful repair of damage, whereby activated fibroblasts undergo the process of apoptosis [28]. Upon uncontrolled activation of fibroblasts in SSc and overproduction of proteoglycans, glycosaminoglycans, fibronectin, and other ECM proteins and factors that further lead to the progressive tissue remodeling, fibroblasts differentiate into contractile and secretory myofibroblasts, which then become the main players in the fibrotic process in all affected organs [15]. Formation of myofibroblasts is a hallmark of SSc, and it was shown that in order to temporarily meet the high demand for these cells due to prolonged tissue damage and/or inflammation, myofibroblast precursors are recruited from multiple sources. Local epithelial and endothelial cells, through the processes of epithelial- or endothelial-mesenchymal transitions, give rise to myofibroblasts, gradually losing their own markers and acquiring new mesenchymal markers [29,30]. One of the sources of myofiroblasts are pericytes, specialized mesenchymal cells, associated with the walls of small blood vessels and smooth muscle cells of blood vessels [31,32]. New myofibroblasts can also arise from fibrocytes, circulating fibroblast precursors of bone marrow origin that migrate to the site of injury/inflammation attracted by signaling molecules. Recent research points out that residual adipocytes can also transdifferentiate into myofibroblasts and thus contribute to the pool of cells that participate in the repair of damaged tissue [33,34]. As a consequence of myofibroblasts activity, tissue damage becomes intensive and irreversible. 

In tandem with the exaggerated production and accumulation of collagen in the skin from SSc patients, altered collagen ultrastructure and biomechanical properties are also apparent [35]. In the skin, not only accumulation but also a more compact organization of collagen fibers and other extracellular matrix proteins in the reticular dermis leads to epidermal thinning, loss of interpapillary ridges, and atrophy of dermal appendages, resulting in some cases to calcinosis, cutaneous ulcers, and skin fibrosis [6].

### 3.1. The Role of Apoptosis in Myofibroblast Survival in SSc

Apoptosis is a physiological type of cell death characterized by the preservation of the plasma membrane integrity, which prevents local inflammatory reactions and tissue damage. Apoptosis plays a key role in emergence and maintenance of SSc [36,37,38,39]. The role of apoptosis in the process of fibrosis was recognized long ago, but the key players and their role in SSc are still under investigation. Due to multi-organ involvement in SSc and the interplay of complex pathways in its initiation and progression, apoptosis of diverse cell types, such as immune cells, endothelial and epithelial cells, myofibroblasts, and fibroblasts have been analyzed in SSc. In the following section, the focus will be on the apoptosis in myofibroblasts as a fundamental player in fibrotic changes in the skin.

#### 3.1.1. TGF-β as a Crucial Molecule in Fibroblast-to-Myofibroblast Transition in SSc Skin

The roles of both intrinsic and extrinsic apoptotic pathways were studied and were shown to be deregulated in the skin of SSc patients [40,41]. Fibroblasts are capable of sensing various stimuli from cell environment, which differs during homeostasis and during pathological processes. In SSc, fibroblasts respond to chemical (humoral factors, such as cytokines, chemokines, growth factors, oxidative stress, hypoxia, epigenetic factors, and non-coding RNAs) and mechanical stimuli (ECM stiffness change), and, as a response to this stimulation, signaling pathways transduce the message inside of the cell [34,42,43,44,45]. A crucial profibrotic cytokine central to development and maintenance of the SSc phenotype is transforming growth factor β (TGF-β) [16,37,46,47]. During fibroblast-to-myofibroblast transition (FMT), TGF-β acts through intracellular pathways, which are known as the canonical SMAD2/3 pathway and the non-canonical TGF-β pathways, inclosing protein kinases ROCK, ERK, and PI3K/AKT. Acting through this molecular network, TGF-β regulates fibroblast proliferation and apoptosis, myofibroblast generation, and ECM protein production [48,49]. As studies about involvement of apoptosis in SSc development showed, both canonical and non-canonical TGF-β pathways are involved in regulation of apoptosis in the skin of SSc patients and experimental models of the disease. Moreover, there is a difference in expression of proteins involved in apoptosis during transition of fibroblasts to myofibroblasts [40,41,50]. 

#### 3.1.2. Autocrine TGF-β Signaling and Death Receptor Apoptotic Pathway

**Figure 1:**
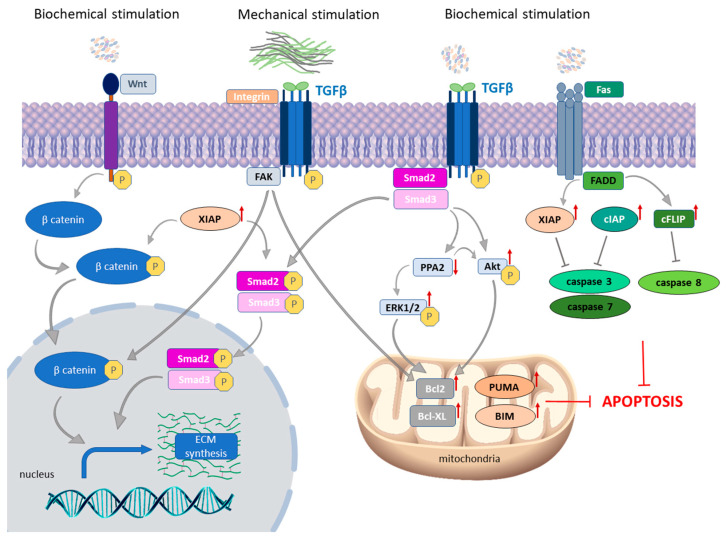
Schematic representation of apoptosis regulation in SSc. In the skin of SSc patients, both intrinsic and extrinsic apoptotic pathways are deregulated. In response to biochemical stimulation, both canonical and non-canonical TGF-β pathways are activated, and XIAP is shown to be a link between them. XIAP also inhibits caspase-3 and 7. As a result of constitutively activated autocrine TGFβ signaling, PP2A level is downregulated in SSc fibroblasts, leading to increased AKT and ERK1/2 phosphorylation. Extrinsic apoptotic pathway is deregulated through cIAP and XIAP proteins. Their higher expression was shown to abrogate caspase-3 and 7 in late-stage fibroblast populations from SSc patients. Matrix stiffness through the activity of fibroblast integrins transmits the mechanical force from the matrix to the actin cytoskeleton through focal adhesion-associated protein FAK. FAK activation and its constitutive phosphorylation of downstream molecules drives profibrotic gene expression. In addition, target genes regulated through this mechanism also include proapoptotic BCL-2 family members Bcl-2, BIM, and PUMA, which activate to prime the cell for apoptosis. In response to this, extensive activation of antiapoptotic Bcl-XL protein takes place, in order to ensure cell survival.

Several studies have demonstrated an autocrine TGF-β signaling in SSc fibroblasts, as a consequence of elevated levels of TGF-β receptors even in the absence of exogenous ligands [51,52,53]. Prolonged activation of TGF-β leads to constitutive activation of AKT and ERK1/2 pathways, which are two major survival pathways that are hyperphosphorylated in SSc fibroblasts and contribute to their resistance to apoptosis [37,54,55,56] (Figure 1). Notably, activation of AKT and ERK1/2 signaling pathways induce the upregulation of the Bcl-2 protein, which is a vital anti-apoptotic protein involved in the mitochondrial apoptotic pathway [57]. In addition, protein phosphatase 2A (PP2A), a main serine/threonine phosphatase responsible for the dephosphorylation of a wide range of signaling molecules, is shown to be downregulated in SSc [58]. In a study by Samuel et al., it was shown that the direct consequence of PP2A downregulation is an increase in ERK1/2 phosphorylation and collagen expression in SSc skin fibroblasts [58]. Importantly, PP2A protein is involved in the death receptor apoptotic pathway, through activation by ceramide, which is included in a wide range of cellular processes, such as cell proliferation, differentiation, and apoptosis [59]. Production of ceramide is highly under the control of lysosomal acidic sphingomyelinase (ASMase), a major sphingolipid enzyme that plays an important role in Fas-mediated apoptosis [60]. ASMase is mediator of the anti-apoptotic effects of TGF-β in dermal fibroblasts, and its deficiency plays a central role in apoptosis resistance of SSc fibroblasts [60]. The effect of ASMase in the survival of SSc fibroblasts was shown to exploit Fas-mediated apoptosis directly through activation of caspase-3 [60].

#### 3.1.3. The Link between Intrinsic and Extrinsic Apoptotic Pathways in SSc Skin

Deregulation of the extrinsic apoptotic pathway was shown to contribute to the resistance of fibroblasts from SSc patients to apoptotic cell death [36,41] (Figure 1). Enrolment of inhibitor of apoptosis protein family members (IAP) in the establishment of the SSc phenotype was shown, specifically cIAP and XIAP. Namely, higher expression of anti-apoptotic proteins cFLIP and cIAP was shown in late-stage fibroblast population from SSc patients, indicating that lesional cells from late-stage patients were more resistant to Fas-induced apoptosis than non-lesional cells from the same patient [41]. cFLIPs is a well-known and efficient anti-apoptotic protein that prevents interaction between adaptor FADD protein and pro-caspase-8, and cIAP is an inhibitor of caspase-3 and -7 activation [61,62]. Examination of the expression of apoptotic suppressor gene *XIAP* showed its upregulation in skin biopsies of SSc patients compared to healthy controls, with particularly high levels in patients with progressive, diffuse cutaneous SSc [50]. XIAP is known to inhibit two members of the caspase family of cell-death proteases, caspase-3 and caspase-7 [63]. Further elucidation of the action mechanism observed in this report revealed that XIAP uses the TGFβ/SMAD3-dependent pathway but also promotes WNT/β-catenin signaling to drive fibroblast activation and tissue fibrosis. These results showed a novel link between two core pathways of fibrosis via XIAP [50]. It was previously shown that WNT/β-catenin signaling has emerged as main pathway of fibrotic tissue remodeling and shown to be hyperactivated in SSc skin biopsies [64,65].

#### 3.1.4. The Role of Mechanical Stimulation on the Expression of Apoptotic Proteins in SSc Fibroblasts

It was shown that FMT and myofibroblast survival depends not only on chemical but also on mechanical signals [66,67]. Elevated mechanical signaling from ECM induces focal adhesion kinase (FAK) activation, and constitutive phosphorylation of FAK in a mechanism that involves integrin b1 drives profibrotic gene expression [68]. Lagares et al. explored detailed molecular mechanisms of this process in dermal fibroblasts derived from patients with SSc and a mouse model of SSc, as well as an in vitro model of mechanical stiffness [40]. Using original BH3 profiling assay to evaluate the sensitivity of fibroblasts to undergo apoptosis (priming) they showed the role of BH3-only activator protein BIM, as well as higher expression of *PUMA*, which belongs to the group of BH3-only apoptotic “sensitizers” acting in direction to prime SSc fibroblasts for apoptotic death [40]. Interestingly, continual mechanical signals from cell surroundings, as a consequence, have upregulation of pro-survival protein B-cell lymphoma-2 extra-large (Bcl-xL), enabling these cells to gain an antifibrotic phenotype. They showed elevated expression of *Bcl-xL* in the cells from SSc patients and in stiffness-primed myofibroblasts in vitro, and they showed that treatment with BH3 mimetic drug ABT-263 induces apoptosis of myofibroblasts both in vitro and in vivo in the bleomycin mouse model of scleroderma [40]. 

### 3.2. The Role of Autophagy in the Progression of Skin Fibrosis in Patients with SSc 

The role of autophagy was investigated in many autoimmune diseases, such as multiple sclerosis, rheumatoid arthritis, psoriasis, systemic lupus erythematosus (SLE), inflammatory bowel diseases, vitiligo, and atopic dermatitis [17,69]. The relevance of autophagy in autoimmune diseases could be analyzed from the point of the immune system, since it is involved in elimination of intracellular pathogens, control of inflammation through inflammatory cytokine secretion, lymphocyte development, antigen presentation and lymphocyte homeostasis, and secretion of immune mediators [17,23]. Due to its role in keratinocyte differentiation and melanocyte survival, autophagy has been examined in various skin disorders, including SLE, psoriasis, vitiligo, infectious skin diseases, and skin cancers [70]. Multiple studies have demonstrated a correlation between dysregulated autophagy and fibrotic diseases, but, despite the existence of shared prognostic markers, both up- and downregulation of autophagy have been hypothesized to be involved in fibrosis progression [21]. Since there is multiorgan involvement in SSc, it could be expected that dysregulation of autophagy could play a role in its pathogenesis and clinical course, and that aberrantly expressed proteins involved in the autophagy could be potential targets for therapy.

**Figure 2:**
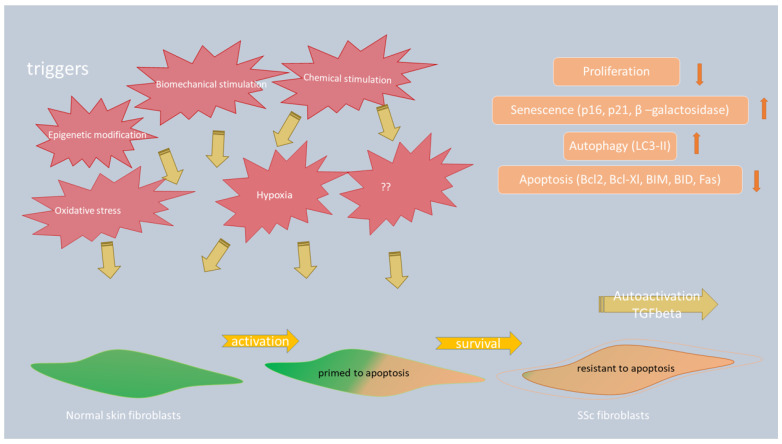
Factors that influence fibroblast-to-myofibroblast transition.

Studies on skin specimens from SSc patients have shown that impaired autophagy contributes to fibrotic phenotype in this disease [71,72,73]. Namely, assessment of key autophagic molecule LC3 by immunofluorescence on skin punch biopsies of SSc patients and control samples showed higher expression of LC3 and increased autophagy in patients compared to healthy controls [71]. This finding is in concordance with results of Mori et al., who described the increased number of intracellular LC3-positive puncta in bleomycin-induced mouse scleroderma skin and in the skin of SSc patients in sclerotic phase. This result reflects autophagy activation and its potential effect on disease progression [73]. Dumit at al. performed proteomics analysis of dermal fibroblasts from healthy subjects of various ages and from patients with SSc, and they showed a lower proliferation rate of aged and SSc fibroblasts measured by minichromosome maintenance (MCM) complex proteins (MCM6 and 7), deregulated autophagy measured by higher expression of LC3 protein and an increased level of senescence measured by increased β-galactosidase activity, and higher p16 and p21 levels [72]. Figure 2 shows the involvement of different mechanisms in fibroblast-to-myofibroblast transition, including autophagy and apoptosis.

## 4. Systemic Sclerosis—Interstitial Lung Disease (SSc-ILD)

Interstitial lung disease associated with systemic sclerosis generally manifests with symptoms such as dyspnea and cough, often accompanied by a non-specific interstitial pneumonia pattern observed on CT scan. However, it is important to note that only a minority of cases meet the criteria for usual interstitial pneumonia [74]. The main pathophysiological characteristics of the SSc-ILD include epithelial injury, dysregulated repair mechanism, activation of immune cells based on the presence of lymphocytes in the alveolar septae and bronchoalveolar lavage, and abnormal fibroblast/myofibroblast function leading to excess collagen deposition [75,76]. This leads to an increase in fibrosis in lungs, which are prone to respiratory failure, and pulmonary complications, which are the most common cause of death in these patients [75]. Moreover, severe restrictive lung disease is present in 13% of patients with SSc [76]. The presence of diffuse cutaneous SSc (dcSSc), older age at the disease onset [77], shorter disease duration [78], male sex [79], and the presence of anti-Scl-70/anti-topoisomerase I antibody and/or absence of anticentromere antibodies are the main risk factors for development and progression of ILD in patients with SSc [77,79].

Fibrotic non-specific interstitial pneumonitis (NSIP) is the predominant pulmonary histological pattern observed in 70% of patients with SSc-ILD, as well as in individuals with other connective tissue disorders, which are responsible for diffuse parenchymal lung disease [80]. A pattern of usual interstitial pneumonia (UIP), which leads to idiopathic pulmonary fibrosis (IPF), is also seen in up to 30% of patients with SSc-ILD [80]. 

**Figure 3:**
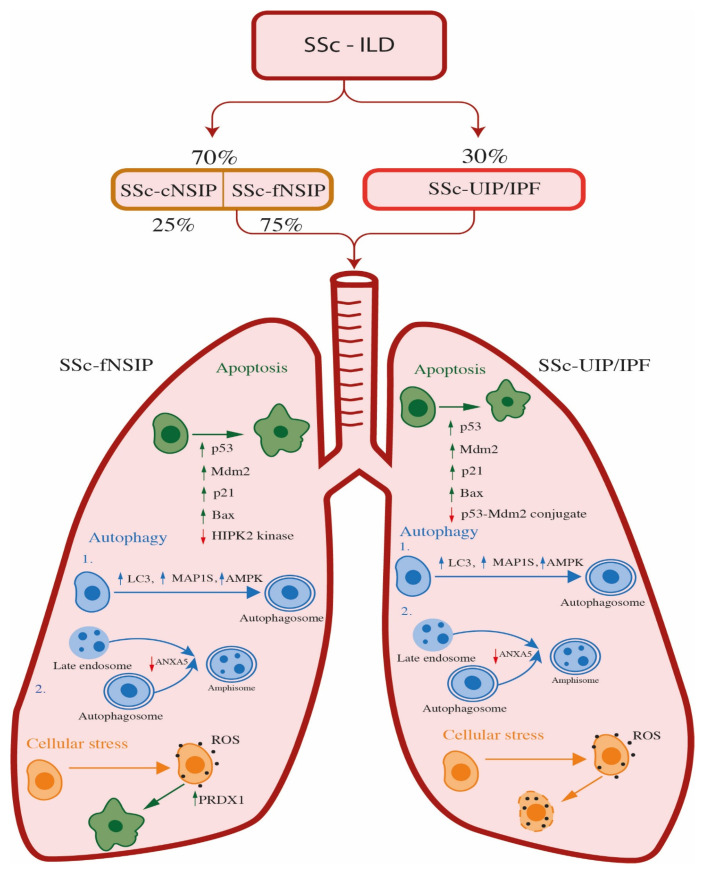
The proposed processes of apoptosis, autophagy, and cellular stress in SSc-ILD patients. Schematic representation of apoptosis, autophagy, and oxidative stress processes in SSc-ILD, where apoptosis is marked in green, autophagy in blue, and oxidative stress in orange. Downregulation of HIPK2 kinase in NSIP and p53-Mdm2 conjugate in UIP/IPF patients indicate the differences between apoptotic pathways involved in the pathogenesis of NSIP and UIP/IPF, respectively. PRDX1 is solely upregulated in NSIP-specific areas in comparison with IPF, suggesting more severe oxidative stress in IPF patients.

NSIP is one of the idiopathic interstitial pneumonia (IIP) classes among idiopathic pulmonary fibrosis, and it lacks the histopathological features of the other subtypes of IIP [81]. NSIP typically involves both lobes of the lungs and shows a predisposition for the lower lobes [82,83,84]. It is characterized by the expansion of the alveolar interstitium due to inflammation and fibrosis. The interstitial changes may be diffuse or patchy, with areas of the lungs appearing normal, but interstitial changes in NSIP are qualitatively uniform, lacking the temporal and regional heterogeneity characteristic of usual interstitial pneumonia. NSIP also lacks the architecturally-distorting scarring or honeycomb change typical of UIP [85]. The inflammatory infiltrate in NSIP is a mixture of plasma cells and lymphocytes. The profusion of plasma cells and lymphoid aggregates with germinal centers is variable but tends to be greater in patients with underlying connective tissue disease, including systemic sclerosis, compared with patients with idiopathic NSIP [86]. Fibroblast foci are rare, and fibrosis tends to consist of dense collagen deposition. The majority of NSIP cases involve both inflammation and fibrosis, but there are cases in which one component predominates, resulting in what is called cellular or fibrotic NSIP (cNSIP and fNSIP). Inflammation of the interstitial cells characterizes the cellular form. The fibrotic form is defined by the thickening and scarring of lung tissue. In SSc patients with NSIP, the fibrotic form is more common than the cellular form, representing more than 75% of all SSc-NSIP cases [87,88] (Table 1, Figure 3).

Patients with systemic sclerosis-associated UIP (SSc-UIP), as well as with other forms of connective tissue disorder (CTD), and CTD-associated UIP, such as rheumatoid arthritis, may have a greater degree of lymphoid hyperplasia encountered on lung biopsy than patients with IPF; however, this finding is not specific. Usual interstitial pneumonia (UIP) is chronic interstitial pneumonia that has a fibrosing course and leads to interstitial expansion that has a preference for the subpleural lung zones and interlobular septa. Lower lobe predominance of chronic interstitial pneumonia, but also the multicolored appearance between microscopic fields, are responsible for the term regional heterogeneity that is often used to describe UIP. Confluent areas of fibrous scarring result in the remodeling of the lung parenchyma in which cystically dilated terminal bronchioles (referred to as honeycomb change) are embedded, resulting in traction of the more proximal airways [89]. Primarily, the occurrence of the UIP pattern was exclusively reserved for IPF; however, this opinion has changed. Furthermore, it is now accepted that the course of the disease in chronic autoimmune diseases with UIP pattern (UIP/AuD), such as systemic sclerosis (SSc-UIP), has a similar course as IPF (UIP/IPF) [90] (Table 1, Figure 3).

### 4.1. The Role of Apoptosis and Oxidative Stress in SSc-ILD Patients

Although the molecular mechanism of apoptosis in SSc-NSIP patients is not described yet, and that apoptosis in NSIP patients without systemic sclerosis is still unknown, it has been shown that the apoptosis of the alveolar epithelial cells type II (AECII) that plays a vital role in UIP/IPF [91] has also been found in NSIP patients [92]. Nakanishi et al. partly covered the topic of the apoptosis and performed immunoblotting and immunohistochemistry analysis of p53, Mdm2, p21, and Bax on lung tissues from IPF and NSIP patients, as well as healthy donors. Wild-type p53 has a role in the suppression of cell growth while the cell tries to repair DNA damage. In cells with DNA damage that cannot be repaired or continue to proliferate, p53 promotes apoptosis. When cells are in a homeostatic state, the levels of p53 protein are very low; however, when cells are in stress, the expression level of p53 is increased [92]. The expression and function of the p53 protein are regulated mainly at the post-transcriptional level, and the main regulator is Mdm2. It mediates the ubiquitination of nuclear and cytoplasmic p53 and induces the degradation of the p53 protein. By targeting p53 for degradation, the Mdm2 inhibits the apoptotic function of p53. The p53 mediates apoptosis by transcriptional activation of pro-apoptotic genes, such as *PUMA*, *Bax*, *Fas*, and *Apaf-1* [93]. After cellular stress, the p53 is upregulated, leading to the transcription of Mdm2. The Mdm2 protein binds to p53, inhibits its transcriptional activity, and promotes nuclear export. On the other hand, phosphorylation of p53 reduces both the p53–Mdm2 feedback loop and nuclear export of p53. The crucial downstream effector of the p53 in cells that contain wild-type p53 after cellular stress is p21. p21 enhances survival in two ways: by promoting DNA repair or by modifying cell death. These findings propose that p21 may be a key regulator of DNA replication and repair after lung injury [92]. The results of Nakanishi and associates revealed that the expression levels of p53, phosphorylated p53, Mdm2, p21, and Bax were upregulated in epithelial cells from patients with IPF and NSIP compared to normal lung parenchyma. Further, levels of p53, phosphorylated p53, Mdm2, p21, and Bax were also significantly increased in IPF patients compared to patients with NSIP. In addition, p53–Mdm2 conjugates were decreased in IPF compared to NSIP and controls. They conclude that cellular stress in both IPF and NSIP patients increases molecules associated with p53-mediated apoptosis and may participate in epithelial cell apoptosis. The reduction of p53–Mdm2 conjugation and p53 degradation in IPF patients may be involved in epithelial cell apoptosis, which may have an impact on poor prognosis in IPF patients compared to NSIP [92]. Korfei et al. performed a comparative proteomic analysis of peripheral lung tissue from IPF patients and fibrotic NSIP patients, while, for a control, they used lungs from 10 organ donors [94]. According to their results, the proteomic profiles of IPF and fNSIP shared the downregulation of anti-apoptotic factors and antifibrotic molecules. However, the proteomic signature of fNSIP was different from IPF in expression levels of proteins involved against oxidative and ER stress and therefore anti-apoptotic strategies. Downregulation of pro-apoptotic p53 kinase HIPK2 (stress-induced homeodomain-interacting protein kinase-2) was observed, and it consequently reduced the level of apoptosis in NSIP compared with IPF patients. This finding can be related to a better prognosis in fNSIP patients [94], most likely due to the normal functioning of the p53-Mdm2 feedback loop since the kinase HIPK2 is inhibited and phosphorylation level is very low; therefore, there are no obstacles for formation of a complex between p53 and Mdm2. However, when comparing those two entities with healthy control samples, HIPK2 was upregulated in both NSIP and IPF.

Oxidative stress is known to disrupt protein folding through the formation of protein carbonyls. The proteomic analysis of antioxidant enzymes peroxiredoxin 1 (PRDX1) was observed to be upregulated in fNSIP and IPF, probably as a response to reported increased oxidative stress in those disorders [95]. Analysis of the cellular distribution in IPF, fNSIP, and lungs from healthy donors discovered that PRDX1 is mainly expressed in ciliated bronchial cells and in alveolar macrophages, and upregulated in groups of patients compared to controls. Prominent induction of PRDX1 in hyperplastic AECII in areas of thickened alveolar septae in some fNSIP lungs was observed. The conclusion was that AECII-localized upregulation of PRDX1 exclusively in NSIP-typical areas such as thickened alveolar septae in fNSIP lungs may represent an attempt of the AECII to regulate the microenvironment in a manner that is beneficial to survival. Furthermore, the expression levels of autophagy markers AMPK, LC3, and MAP1S between groups of patients with UIP/IPF and UIP/AuD were equal, according to the staining pattern. Nevertheless, analyzed autophagy markers were overexpressed in both groups of patients when compared to the control group regardless of the etiology of the UIP pattern, most likely as the answer of epithelial cells and myofibroblasts to local hypoxia. Analysis of antioxidant proteins revealed overexpression in NSIP patients in contrast to IPF, indicating that better outcome and survival in NSIP patients in comparison to IPF patients is due to better performance of reparative mechanism against ROS and misfolded protein carbonyls, and that fNSIP and IPF can be distinguished on the molecular level by analysis of proteins involved in oxidative and ER stress [94] (Table 2, Figure 3).

### 4.2. The Role of Autophagy in SSc-ILD Patients

#### 4.2.1. The Role of Autophagy in SSc-ILD Patients with UIP Pattern

Two processes that seem to have a crucial role in the development of UIP/IPF are senescence and autophagy [97,98]. Senescence is characterized by the cell-cycle arrest in the G1 or possibly G2 phase, which prevents the proliferation of damaged cells [99,100], and it is initiated by cyclin-dependent kinase inhibitors such as p16, p21, and p53. Cellular senescence can be induced by DNA damage, telomere shortening/dysfunction, activation of oncogene or loss of tumor suppressor functions, epigenetic changes, and organelle damage [101]. Mediators of senescence, most prominently, IL-1, IL-6, IL-10, and TGF-β, cause a prolonged repair process, finally resulting in fibrosis, and may play a role in the epithelial-mesenchymal transition in UIP, which may be brought on by dysfunctional autophagy [98,102,103]. 

Autophagy is a cellular process, which, in order to promote homeostasis, differentiation, development, and survival, eliminates molecules and subcellular elements via lysosomal degradation, and, under specific conditions (hypoxia, starvation, or the absence of growth factors), is considerably increased [104]. Autophagy markers that can be evaluated in UIP/IPF are: adenosin-5′ monophosphate-activated kinase (AMPK), the activator of autophagy; microtubule-associated protein 1A/1B-light chain 3 (LC3); and microtubule-associated protein 1S (MAP1S), involved in the development and degradation of autophagosomes [105,106,107]. Gallob et al. compared the autophagy and senescence markers between patients with UIP/IPF and UIP/AuD. All analyzed autophagy markers were overexpressed in both groups of patients when compared to a control group in epithelial cells within the remodeled areas and in myofibroblasts, regardless of the etiology of the UIP pattern. However, they found that there were no differences in the expression of autophagy markers between groups of patients according to the staining pattern. The authors concluded that the upregulation of autophagy is possibly the answer of epithelial cells and myofibroblasts to local hypoxia [90]. 

#### 4.2.2. The Role of Autophagy in SSc-ILD Patients with fNSIP Pattern

To the best of our knowledge, there are no scientific papers addressing the role of autophagy in patients with SSc-NSIP. However, there are only a few scientific publications addressing autophagy in patients with non-specific interstitial pneumonia, more specifically in fNSIP, which is predominantly present in systemic sclerosis patients with lung involvement. Korfei and coworkers compared the proteome of lung tissue from IPF and fNSIP patients, relative to control lung tissue, and they identified that among downregulated proteins in both groups of patients was one autophagy protein, annexin A5 (ANXA5) [94]. Ghislat et al. found that annexin A5, a Ca^(2+)^-dependent phospholipid-binding protein, was increased on lysosomal membranes under starvation, suggesting a role of this protein in starvation-induced lysosomal degradation. Experiments with transient over- and underexpression showed that annexin A5 increased lysosomal protein degradation, mainly as the result of inducing autophagosome–lysosome fusion, but not receptor-mediated endocytosis. Further, their results indicated that, under the low levels of annexin A5, there was an increase in autophagosome–late endosome (with proteolysis competence) fusion to form amphisomes. Consequently, the authors propose a dual-acting role of annexin A5 in regulating the endocytic and autophagic pathways and the fusion of autophagosomes with lysosomes and endosomes, and they conclude that ANXA5 is a positive regulator of autophagy and a negative regulator of endocytosis [96]. Since proteome analysis was performed on samples derived from patients with IPF and fNSIP and a decreased expression of ANXA5 protein was detected in both groups of patients, autophagosome–late endosome fusion to form amphisomes (convergence of endocytic and autophagy pathways) can be proposed as the role of the ANXA5 protein in the process of autophagy in those disorders (Table 2, Figure 3).

## 5. Conclusions

In this paper, we summarized the role of apoptotic and autophagic markers specifically in the skin and lungs of SSc patients. Many studies, including multiomic studies [108,109,110], have shown differential expression of molecular markers in different tissues, making it clear that much more effort is needed to elucidate the control mechanisms fundamental to the development and progression of this disease. Thanks to the acquired knowledge about its pathogenesis, some manifestations of the disease, such as scleroderma renal crisis, pulmonary arterial hypertension, digital ulceration, and gastro-esophageal reflux, are now treatable, but there are various complications associated with systemic sclerosis that still represent a challenge for treatment [111]. 

By reviewing the literature on the markers of apoptosis, autophagy, and oxidative stress in patients with SS-ILD (SSc-fNSIP and SSc-UIP/IPF), we can conclude that these mechanisms are poorly described and are almost completely unknown in this disease. There are only literature data on patients with fNSIP and UIP/IPF pulmonary manifestations in other connective tissue diseases, as well as in autoimmune inflammatory diseases. Thus, the proposed mechanisms are only hypothesized, and it would be necessary to use novel methods, such as liquid biopsy, in order to determine which markers are aberrantly regulated in SSc patients. Knowing the molecular biomarkers involved in the autophagy, apoptosis, oxidative stress, and other processes relevant for SSc could allow their detection from the circulation. This approach would enable non-invasive, early, and precise diagnosis, and would be one of the factors that would contribute to lower mortality from this rare disease.

When it comes to skin manifestations in SSc, better knowledge of the markers involved in the processes of apoptosis and autophagy could facilitate the determination of the stage of the disease and contribute to a more precise classification of patients. Moreover, determining the expression of these genes in each patient could enable the development of the next generation of biological therapeutics that could lead to individualization of the therapy.

## 6. Limitations of the Study

This is a narrative literature review focused on summarizing data on the role of autophagy and apoptosis processes in the skin and lungs of SSc patients. Therefore, this study is prone to the limitations of narrative research methodology, such as selection bias, difficulty in determining complex interactions, and limitation in drawing accurate conclusions. To prevent this, the authors strictly adhered to the inclusion and exclusion criteria, tried to present the data objectively, and tried to include publications with conflicting results, in order to reach more accurate conclusions, without interpretation.

## Figures and Tables

**Table 1:** Comparison of histologic features in SSc-ILD patients.

	SSc-ILD		Control Lung Donors (CLD)	Ref.
	NSIP	UIP/IPF		
Histologic Features				
Distribution of fibrosis	Uniform	Regional heterogeneity	Absent	[84]
Localization of fibrosis	Lower lobes	Lower lobes	Absent	[89]
Honeycomb change	Absent	Present	Absent	[85]

**Table 2:** Markers involved in apoptosis, autophagy, senescence, and oxidative stress processes in ILD patients.

	ILD		Control Lung Donors (CLD)	Ref
	NSIP	UIP/IPF		
Apoptosis				
p53	Upregulated when compared with CLD Downregulated when compared with UIP/IPF	Upregulated	Homeostasis	[92,93]
Phosphorylated p53				
Mdm2				
p21				
Bax				
p53-Mdm2 conjugate	-	Downregulated	Homeostasis	
HIPK2 kinase	Downregulated	-	Homeostasis	[94]
Autophagy				
LC3-II	Upregulated	Upregulated	Homeostasis	[90]
MAP1S	Upregulated	Upregulated		
pAMPK	Upregulated	Upregulated		
ANXA5	Downregulated	Downregulated		[94,96]

## Data Availability

Not applicable.
